# Relationship between Willingness and Psychological Characteristics of Suicide Prevention Telephone Counselors: A Retrospective Observational Study

**DOI:** 10.3390/ijerph18189800

**Published:** 2021-09-17

**Authors:** Osamu Murakami, Kanae Kanda, Nlandu Roger Ngatu, Tomohiro Hirao

**Affiliations:** Department of Public Health, Faculty of Medicine, Kagawa University, 1750-1 Ikenobe Miki-cho Kita-gun, Kagawa 761-0793, Japan; kanda.kanae@kagawa-u.ac.jp (K.K.); ngatu@kagawa-u.ac.jp (N.R.N.); hirao.tomohiro@kagawa-u.ac.jp (T.H.)

**Keywords:** telephone counselor, willingness, grit, mental health, physical health, self-efficacy, social support

## Abstract

Suicide is a major public health issue worldwide, and telephone counseling is an important preventive measure. As the number of telephone counselors is insufficient in Japan, public needs cannot be fully met. Willingness is important for securing telephone counselors, but few studies have examined the willingness to engage in telephone counseling activities. Therefore, we investigated the relationship between telephone counselors’ willingness to perform their activities and their psychological characteristics, health status, and received social support. In this study, a questionnaire survey was conducted by mail among telephone counselors belonging to the Federation of Inochi No Denwa in Japan. The total number of valid responses was 709 (recovery rate: 50.4%). Following an exploratory factor analysis, three factors were extracted: (1) willingness to engage in telephone counseling activities, (2) sense of being burdened by telephone counseling activities, and (3) sense of difficulty in coping. Structural equation modeling, using all the factors, showed that social support and grit were directly related to the willingness to engage in telephone counseling activities, while physical health, mental health, and general self-efficacy were indirectly related to it. The findings obtained may be useful in devising concrete measures for telephone counselors to continue their activities.

## 1. Introduction

With more than 700,000 deaths per year worldwide, suicide is a major public health issue. The World Health Organization (WHO) has stated that reducing suicide-related mortality is a “global responsibility” [1,2]. Although the number of suicides per 100,000 population in Japan has been on a downward trend since peaking at 27.0 in 2003, having fallen to 16.7 in 2020 [3], it is still a serious social problem. Telephone counseling is one approach to preventing suicide. Organizations that provide this service were established in the United Kingdom in the 1950s, and today, volunteer counselors with prescribed training provide telephone counseling in many countries [4]. It can reduce isolation and enhance social support for people at risk of suicide. Therefore, the provision of telephone counseling is an important tool for determining suicide rates [5,6]. In Japan, 24-h telephone counseling centers have been established throughout the country by the Federation of “Inochi no Denwa” (FIND), playing an important role in suicide prevention.

The number of telephone counseling sessions in Japan is large, even with the declining suicide rates, indicating the persistence of the demand for such services [7]. However, the number of telephone counselors is chronically small because all of them are volunteers. Furthermore, the response rate to incoming calls is very low, implying that they are not able to adequately respond to requests [8]. In addition, it is difficult to secure counselors to work late at night (10 p.m. to 6 a.m.) in a 24-h service. To solve this shortage, it is necessary to acquire and train new counselors, as well as take measures to prevent them from leaving the service by improving the working condition of those working the night shift [9].

In a previous study, surrounding support for volunteers and their perceived burden of activities affected their willingness to engage in counseling [10]. In addition, people with higher grit and self-efficacy were more likely to continue working on difficult tasks [11,12,13]. Furthermore, it has been reported that healthy volunteers have a longer activity period and less avoidance behavior [14], and workers in good health have a higher willingness to continue working after retirement [15]. In telephone counseling, willingness is important for volunteers to participate and continue their activities; however, few studies have examined factors related to the willingness to engage in telephone counseling and the relationship between them.

**Figure 1:**
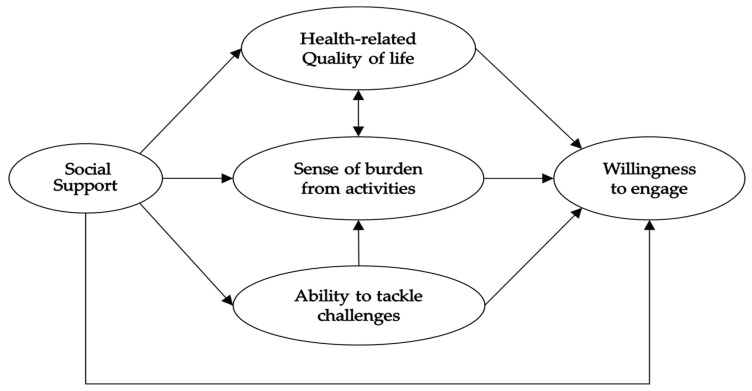
Analytical model.

Therefore, we hypothesized (Figure 1) that (1) the willingness to engage in telephone counseling increases when the “health-related quality of life” is favorable, the “sense of burden from activities” is low, and the “ability to tackle challenges” is high, and (2) a situation with high “social support” will have a positive impact on each factor and increase willingness.

The purpose of this study was to explore and examine the factors that enhance the willingness to engage in telephone counseling activities for suicide prevention.

## 2. Materials and Methods

### 2.1. Target Population and Data Collection

A questionnaire survey was conducted, targeting telephone counselors who belonged to suicide prevention centers in Japan (FIND). We asked 23 centers, nationwide, that provide FIND’s 24-h telephone counseling service to cooperate in the survey, and received consent from 10 centers. Therefore, 1408 sets of questionnaires (questionnaire booklets, implementation instructions, and return envelopes) were sent to 10 centers in response to their requests. The questionnaires were then distributed individually to counselors at each center. The questionnaire was anonymous, and the provision of responses was left to the counselors’ choice. The completed questionnaires were individually mailed to Kagawa University. The survey was initially planned to be conducted from 1 February to 31 March 2020; however, considering the influence of COVID-19, the deadline was extended to 31 July 2020.

### 2.2. Sample-Size Calculation

The sample size was calculated based on the physical component (PCS) SF-8 summary score after reviewing the relevant literature for each rating scale [16]. The effect size was 2.8; standard deviation, 7.24; significance level, 0.05; and power, 0.8. The calculated sample size was 212. Based on the results, the required sample size was set to 250. Assuming a 40% response rate, the minimum number of copies required for distribution was calculated to be 625.

### 2.3. Contents of the Questionnaire

To evaluate the willingness to engage in telephone counseling activities, 26 questions on “thoughts on activities” were prepared (Table 1). These questions were developed based on the questionnaire contents used in other researchers’ studies [8,9,10] and a preliminary survey of counselors. A four-point Likert scale was used as the response method, including “completely agree”, “somewhat agree”, “somewhat disagree” and “completely disagree.” Regarding telephone counseling activities during the night shift, 20 questions asked participants whether they had experience working the night shift, the number of years they had been worked night hours, reasons for taking the night shift, and problems while on the night shift, using a 4-point Likert scale.

The Japanese version of the Grit-S [17] was used to measure grit; grit refers to the ability to respond to and achieve long-term goals through passion (consistency of interest) and perseverance (consistency of effort), which are necessary for sustained effort. Self-efficacy is the degree of confidence and conviction in one’s ability to perform and accomplish the necessary actions to produce results for a given task; it was measured through the Japanese version of the General Self-Efficacy Scale (GSES) [18]. The Japanese version of the Multidimensional Scale of Perceived Social Support (MSPSS) [19] was used to measure social support, which refers to the emotional and material support exchanged in social relationships. For health-related quality of life (QOL), the Japanese version of the Short-Form 8-Item Health Survey (SF-8) [20] was used to measure the physical component summary (PCS) and mental component summary (MCS). Grit and general self-efficacy were used to assess the ability to tackle challenges. Gender, age, family members living together, employment status, telephone-counseling-activity status, and licenses or certificates were asked as basic information.

### 2.4. Data Analysis

Descriptive statistics were used to assess age, family-living-together, employment status, telephone-counseling-activity status, and qualifications. An exploratory factor analysis was conducted to find potential common factors in the “thoughts on telephone counseling activities,” and the reliability of each factor was evaluated using Cronbach’s alpha coefficient. Structural equation modeling was used to identify the causal relationships between variables. The model fit was assessed using the comparative fit index (CFI) and root mean square error of approximation (RMSEA) indices. To compare each factor and the night shift task, a one-way analysis of variance (Welch’s method) was conducted. The significance level was set at 5% (two-tailed). JMP Pro 15 (SAS Institute Inc., Cary, NC, USA) and SPSS Statistics 25 (IBM, Armonk, NY, USA) were used for the statistical analyses.

### 2.5. Ethics Statement

This study was approved by the Ethics Committee of the Kagawa University Graduate School of Medicine (No. 2019-205). The answers to all questionnaires were voluntary and anonymous. Respondents were asked to mark their consent to their participation in the survey.

## 3. Results

The total number of collected questionnaires was 710 (recovery rate: 50.4%), of which 709 were used for analysis after excluding missing data from the required items. The attributes of the subjects and scores for each scale are shown in Table 2. Of all the subjects, 138 were men (19.5%) and 569 women (80.3%). The most common age group for both genders was their 60s, accounting for about half of the respondents, and most lived with their spouses. In terms of employment status, more than half of the men were full-time employees or retired, while more than half of the women were occupied by housekeeping or worked part-time. The highest percentage of respondents (both men and women) had been active for nine years or less. There was a wide range of qualifications, but most participants were in healthcare or teaching. The status of the counselors in charge of midnight shifts is shown in Table 2.

The scores of Grit-S, GSES, MSPSS, SF-8 PCS, and SF-8 MCS were similar to those in previous studies [19,20,21,22], and there was no specific trend within the survey subjects. In the MSPSS, the scores were significantly higher for women, a trend that has also been observed in prior research [19].

Table 3 shows the results of the analysis of the questionnaire item “thoughts on telephone counseling activities.” Following the factor analysis, 16 items representing 3 factors were extracted from 26 items (extraction method: maximum likelihood method; rotation method: Promax, factor loading threshold: 0.40). Based on the tendency shown by the content of the items in each factor, we named the first factor “willingness to engage in activities,” the second factor “sense of burden from activities,” and the third factor “sense of difficulty in coping.” For internal consistency, Cronbach’s *α* coefficient for each factor was calculated at 0.82, 0.82, and 0.75, respectively, confirming the high reliability of each factor.

Correlation coefficients were calculated to confirm the relationship between the willingness to engage in activities (WtE) and each factor (Table 4). A negative correlation was found between WtE and sense of burden from activities. A positive correlation was found between the latter and the sense of difficulty in coping. Weak positive correlations were found between Grit-S and GSES, Grit-S and WtE, MSPSS and WtE, and GSES and SF-8 MCS. Weak negative correlations were found between GSES and sense of burden from activities, GSES and sense of difficulty in coping, SF-8 MCS and sense of burden from activities, as well as between WtE and a sense of difficulty in coping.

**Figure 2:**
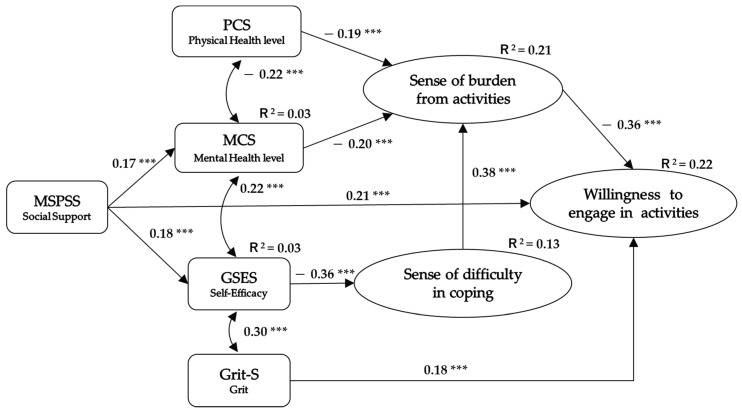
Relationship among factors related to willingness to engage in activities. **Note:** Arrows indicate regressions; numbers are standard partial regression coefficients. Double-headed arrows indicate correlations; numbers are partial correlation coefficients. R^2^: coefficient of determination. *** *p* < 0.001.

Structural equation modeling was conducted to identify the causal relationships between the variables (Figure 2). The analysis was as follows: WtE was set as the objective variable, and the explanatory variables were the sense of burden from activities, sense of difficulty in coping, MSPSS, Grit-S, GSES, SF-8 PCS, and SF-8 MCS. The path diagram shows a high degree of fit (CFI = 0.958, RMSEA = 0.049), with grit having a direct effect on WtE (standard partial regression coefficient *β* = 0.18, *p* < 0.001). Social support had a direct effect on WtE (*β* = 0.21, *p* < 0.001), and an indirect effect by affecting mental health and self-efficacy (*β* = 0.02). General self-efficacy had an indirect effect on WtE by affecting the sense of difficulty in coping (*β* = 0.05), and physical and mental health had an indirect effect on WtE by affecting the sense of being burdened by activities (*β* = 0.07/0.07). The coefficient of determination (R^2^) for WtE in this model was 0.22 (*p* < 0.001), which means each factor explained 22% of the variation in participants’ WtE.

Regarding the influence of the night shift, significant differences were found in the sense of difficulty in coping and SF-8 PCS (Table 5). The “currently in charge of the night shift” group tended to have higher physical health and lower sense of difficulty in coping.

## 4. Discussion

The purpose of this study was to extract the factors that constitute the willingness to engage in telephone counseling activities and to examine the relationships among those factors.

The results revealed that social support was the factor most related to willingness to engage in telephone counseling. The path diagram showed that social support had both a direct and an indirect effect on willingness to engage in telephone counseling activities, the latter by affecting mental health and self-efficacy. Previous studies have reported that those with high social support have better mental health than those with low social support [23], while the prevention of emotional exhaustion and promotion of a healthy social work climate may support the willingness to work [24]. While the social support received in telephone counseling activities is expected to be multifaceted, including from organizations, co-workers, family, and others, this study focused on support from the organization and co-workers, examining what measures the organization can take to ensure the continuation of its counselors’ activities.

Grit was the second factor related to the willingness to engage in telephone counseling activities. Winkler et al. [11] reported that in strict special military instruction, members with high grit were more likely to complete their training, and salesclerks with high grit were less likely to quit their jobs. In this study, grit had a direct effect on WtE and showed a positive correlation, suggesting that high grit is a factor that improves the willingness of telephone counselors to engage their work and motivates them to continue counseling.

Similar to grit, self-efficacy is an important indicator for evaluating the ability to tackle challenges. In this study, a negative correlation was found between self-efficacy and the sense of difficulty in coping, and the path diagram showed an indirect effect on the willingness to engage in telephone counseling activities. Devarajooh et al. [12] found that high self-efficacy facilitated the practice of difficult self-care in diabetes, and Kawamura et al. [13] reported that a diet program enhanced self-efficacy, which promoted changes in health behavior. These findings suggest that self-efficacy is also a factor in the continuation of activities.

Therefore, it can be inferred that enhancing social support and the ability to tackle challenges will be effective in increasing willingness to engage in telephone counseling activities. For this purpose, it is necessary to develop specific strategies. The following studies provided specific strategies: Duckworth et al. [25] showed that the grit of students is fostered and enhanced when the goal structure in schools is more acquisition-oriented, which emphasizes the value of learning, rather than high-achievement-oriented. Mirza et al. [26] reported that the use of five thematic strategies in the educational curriculum of medical students, namely “planning,” “metacognitive skills,” “mastery-based learning,” “cognitive strategies,” and “self-regulation,” significantly increased grit, improved academic performance, and reduced the number of dropouts. Tompkins [27] described strategies to increase the supervisee’s self-efficacy in cognitive therapy based on four items: “performance accomplishment” as a successful experience, “vicarious experience” through observation, “persuasion” through suggestion and instruction, and “physiological states” through the perception of physiological responses. Incorporating these strategies into the training of telephone counselors may increase the effectiveness of such programs.

Finally, the results of the analysis of the night shifts are discussed. In this study, a comparison of the three groups, according to their night-shift status, showed significant differences in physical health and the sense of difficulty in coping. Those in charge of night shifts had higher levels of physical health, while those who had worked night shifts in the past had lower levels. In addition, those in charge of night shifts had less sense of difficulty in coping, while those who had never worked night shifts had more. Based on these results, two issues were considered. First, counselors with no night shift experience tend to have more difficulty in coping, which may inhibit their willingness to choose these shifts. The second issue is that counselors who quit night shifts tend to have low physical-health levels, which may inhibit their willingness to adopt a physically demanding schedule. At Mental Health Helplines in New Zealand, the implementation of a high-quality training program is regarded as useful, not only for improving consultation skills but also for personal growth and self-care. In addition, it has been reported that such organizational support creates a sense of belonging and is effective for sustaining activities [10]. It has also been pointed out that proper shift scheduling is important in preserving the health of workers [28]. Therefore, to address these issues, it is important to improve response skills through training sessions, and to set up an appropriate schedule that considers the health condition of each counselor when planning the assignment.

This study had several limitations. First, it was a cross-sectional study, and the time axis was not considered, therefore it was not possible to make definitive statements regarding causality. Second, counselors who responded may have been more active in telephone counseling than those who did not, and the low response rate to the questionnaire may have biased the results. Third, as this study was conducted from the perspective of volunteer activities, the questionnaire mainly consisted of questions about the counselors, and no reference was made to the organization.

Thus, the future challenge is to develop specific strategies to motivate telephone counselors and promote their continuation as counselors based on the findings of this study. As specific support measures for telephone counselors, we would like to suggest considerations of (1) improving the contents of various training programs, (2) supporting the planning of appropriate responsibilities, based on an understanding of each counselor’s situation, and (3) creating opportunities for staff and counselors to interact with each other.

In addition, the factors discussed in this study were able to explain 22% of the willingness to engage in activities in telephone counseling; however, exploring other determinants is an interesting topic for future research.

## 5. Conclusions

This study revealed that social support and grit have a significant impact on the willingness to engage in telephone counseling activities. In addition, psychological and health factors were also found to be interrelated, influencing the sense of being burdened by activities and that of difficulty in coping, consequently playing an important role in the willingness to engage in activities.

By clarifying the relationship between these factors, it is possible to suggest concrete measures for continuing telephone counselors’ activities.

## Figures and Tables

**Table 1:** Questions on “thoughts on activities”.

Items	
1	Activities lead to my personal growth.
2	I am able to help the people I consult with.
3	I am able to participate in society.
4	I am able to build good relations with my colleagues.
5	Telephone counseling activities fulfill me and are rewarding.
6	My interests and activities are aligned.
7	I would like to continue telephone counseling activities.
8	I would like to improve myself through independent study and training sessions.
9	I am willing to adjust my daily life for telephone counseling activities.
10	I am satisfied with telephone counseling activities.
11	I would recommend participate in telephone counseling activities to my friends if they wanted to.
12	I would like to increase my allotted time.
13	The organization’s system and management are appropriate.
14	Physical burden is high.
15	Mental burden is high.
16	Economic burden is high.
17	Time burden is high.
18	My competence as a telephone counselor is insufficient.
19	The rhythms of my life are disrupted.
20	Commuting to the center is a burden.
21	Dealing with serious issues such as suicide is difficult.
22	Dealing with sexual counseling is difficult.
23	Dealing with frequent callers is difficult.
24	Balancing work (schoolwork) and housework with activities is difficult.
25	Gaining understanding and cooperation from family is difficult.
26	It would be desirable to be compensated for my telephone counselling activity.

**Table 2:** Characteristics of participants and scores for scales.

Variables	Male (*n* = 138)		Female (*n* = 569)		Total (*n* = 707)	
	*n*	%	*n*	%	*n*	%
Age (years)						
Over 80	2	1.4	8	1.4	10	1.4
70–79	37	26.8	132	23.2	169	23.9
60–69	73	52.9	286	50.3	359	50.8
50–59	22	15.9	111	19.5	133	18.8
40–49	4	2.9	27	4.7	31	4.4
30–39	0	0	5	0.9	5	0.7
Total	138	100	569	100	707	100
Family members living together						
(Multiple answers)						
Spouse	113	95	390	89.9	503	91
Children	17	14.3	93	21.4	110	19.9
Parents	17	14.3	45	10.4	62	11.2
Grandparents	2	1.7	2	0.5	4	0.7
Total respondents	119	100	434	100	553	100
Employment status						
Full-time	38	27.7	89	15.9	127	18.2
Self employed	27	19.7	36	6.4	63	9
Part-time	17	12.4	162	28.9	179	25.6
Housekeeping	0	0	165	29.4	165	23.6
Student	0	0	1	0.2	1	0.1
Retired	41	29.9	83	14.8	124	17.8
Other	14	10.2	25	4.5	39	5.6
Total	137	100	561	100	698	100
Experience as a telephone counsellor (years)						
Over 30	5	3.6	21	3.7	26	3.7
25–29	4	2.9	33	5.9	37	5.3
20–24	10	7.2	51	9	61	8.7
15–19	8	5.8	79	14	87	12.4
10–14	20	14.5	114	20.2	134	19.1
5–9	45	32.6	134	23.8	179	25.5
Under 4	46	33.3	132	23.4	178	25.4
Total	138	100	564	100	702	100
Licenses or certicates (Multiple answers)						
Healthcare	21	15.2	113	19.9	134	19
Education	23	16.7	145	25.5	168	23.8
Others	32	23.2	144	25.3	176	24.9
Total respondents	138	100	569	100	707	100
State in charge of midnight shift	Currently In Charge		Past In Charge		No Experience of Being In Charge	
	* n *	%	* n *	%	* n *	%
	393	57.7	137	20.1	151	22.2
Scores for scales	Male		Female		Total	
	mean	SD	mean	SD	mean	SD
Grit-S	3.42	0.67	3.45	0.63	3.45	0.64
GSES	9.82	4.17	9.15	3.94	9.3	4
MSPSS	4.97	1.11	5.31	1.04	5.25	1.06
SF-8 PCS	49.6	7.37	49	7.36	49.06	7.36
SF-8 MCS	48.33	7.68	48.2	6.98	48.19	7.11

Note: Two respondents did not answer the gender question; therefore, the total of gender responses was 707. Abbreviations: GSES, General Self-Efficacy Scale; MSPSS, Multidimensional Scale of Perceived Social Support; SF-8, Short-Form 8-Item Health.

**Table 3:** Factor analysis on “thoughts on telephone counseling activities”.

Items	Factor Loading		
	Willingness to Engage in Activities	Sense of Burden from Activities	Sense of Difficulty in Coping
Telephone counseling activities fulfill me and are rewarding.	0.784	0.019	−0.024
My interests and activities are aligned.	0.712	0.006	−0.002
I would like to continue telephone counseling activities.	0.697	−0.029	−0.030
I am satisfied with telephone counseling activities.	0.601	−0.114	−0.126
I would like to improve myself through independent study and training sessions.	0.598	0.013	0.123
I am willing to adjust my daily life for telephone counseling activities.	0.568	0.083	0.071
Time burden is high.	−0.027	0.792	−0.013
Physical burden is high.	0.053	0.709	−0.068
Rhythm of life is disrupted.	−0.023	0.658	−0.062
Balancing work (schoolwork) and housework with activities is difficult.	−0.064	0.640	0.043
Mental burden is high.	0.009	0.596	0.173
Economic burden is high.	0.065	0.559	−0.017
Dealing with frequent callers is difficult.	0.056	−0.050	0.740
Dealing with sexual counseling is difficult.	−0.048	−0.032	0.732
Dealing with serious cases such as suicide is difficult.	0.072	0.042	0.611
My competence as a telephone counselor is insufficient.	−0.024	0.026	0.551
Cronbach’s coefficient α	0.82	0.82	0.75

Note: Extraction method: maximum likelihood method; rotation method: Promax, factor loading threshold: 0.40.

**Table 4:** Correlations among factors related to willingness to engage in activities.

Factor	Grit-S	MSPSS	GSES	SF-8 PCS	SF-8 MCS	Willingness to Engage in Activities	Sense of Burden from Activities	Sense of Difficulty in Coping
Grit-S	-							
MSPSS	0.062	-						
GSES	0.329 ***	0.191 ***	-					
SF-8 PCS	−0.022	0.076 *	0.101 **	-				
SF-8 MCS	0.113 **	0.154 ***	0.262 ***	−0.185 ***	-			
Willingness to Engage in Activities	0.247 ***	−0.256 ***	0.165 ***	0.055	−0.127 **	-		
Sense of Burden from Activities	−0.163 ***	−0.102 **	−0.216 ***	−0.183 ***	−0.224 ***	−0.404 ***	-	
Sense of Difficulty in Coping	−0.179 ***	−0.032	−0.362 ***	−0.070	−0.162 ***	−0.240 ***	0.416 ***	-

Note: *** *p* < 0.001, ** *p* < 0.01, * *p* < 0.05.

**Table 5:** Relationship between the experience of night shift and factors related to willingness to engage in activities.

Factor	Currently in Charge (*n* = 393)		Past in Charge (*n* = 137)		No Experience of Being in Charge (*n* = 151)		*p* Value	Partial *η*^2^
	Mean	SD	Mean	SD	Mean	SD		
Grit-S	3.46	0.64	3.42	0.68	3.46	0.63	0.824	0.001
GSES	9.52	3.99	8.79	4.21	9.27	3.74	0.232	0.005
MSPSS	62.6	12.8	64.5	11.9	62.1	13.6	0.252	0.004
SF-8 PCS	49.7	6.6	47.6	8.4	48.8	7.8	0.027	0.013
SF-8 MCS	48.5	7.1	47.4	7.8	48.0	6.8	0.301	0.004
Willingness to Engage in Activities	0.001	0.940	0.070	0.898	−0.096	0.879	0.283	0.004
Sense of Burden from Activities	0.021	0.936	0.082	0.903	−0.029	0.909	0.593	0.002
Sense of Difficulty in Coping	−0.111	0.918	0.081	0.801	0.157	0.819	0.002	0.018

Note: Welch’s test was used for the one-factor analysis of variance. Partial *η*^2^: Effect size (partial correlation ratio). The values of “willingness to engage in activities.” “sense of burden from activities,” and “sense of difficulty in coping” are the factor scores.
